# Commentary: childhood cancer near nuclear power stations

**DOI:** 10.1186/1476-069X-8-43

**Published:** 2009-09-23

**Authors:** Ian Fairlie

**Affiliations:** 1 115 Riversdale Road, Highbury, London N5 2SU, UK

## Abstract

In 2008, the KiKK study in Germany reported a 1.6-fold increase in solid cancers and a 2.2-fold increase in leukemias among children living within 5 km of all German nuclear power stations. The study has triggered debates as to the cause(s) of these increased cancers. This article reports on the findings of the KiKK study; discusses past and more recent epidemiological studies of leukemias near nuclear installations around the world, and outlines a possible biological mechanism to explain the increased cancers. This suggests that the observed high rates of infant leukemias may be a teratogenic effect from incorporated radionuclides. Doses from environmental emissions from nuclear reactors to embryos and fetuses in pregnant women near nuclear power stations may be larger than suspected. Hematopoietic tissues appear to be considerably more radiosensitive in embryos/fetuses than in newborn babies. Recommendations for advice to local residents and for further research are made.

## Introduction

Increased incidences of childhood leukemias were first reported near UK nuclear facilities in the late 1980s. Various explanations were offered for these increases; however the UK Government Committee on the Medical Aspects of Radiation in the Environment (COMARE) concluded in a series of reports [1-4] that the causes remained unknown but were unlikely to involve radiation exposures. This was mainly because the radiation exposures from these facilities were estimated to be too low, by two to three orders of magnitude, to explain the increased leukemias.

Recently, the KiKK (Kinderkrebs in der Umgebung von KernKraftwerken = Childhood Cancer in the Vicinity of Nuclear Power Plants) study [5,6] has rekindled the childhood leukemia debate. The KiKK study had been established partly as a result of an earlier study by Körblein and Hoffmann [7] which had found statistically significant increases in solid cancers (54%), and in leukemia (76%) in children aged < 5 within 5 km of 15 German NPP sites. It reported a 2.2-fold increase in leukemias and a 1.6-fold increase in solid (mainly embryonal) cancers among children living within 5 km of all German nuclear power stations. The web publication [8] of the study in December 2007 resulted in a public outcry and media debate in Germany which has received little attention elsewhere.

The KiKK case-control study commands attention for a number of reasons. The first is its large size: it examined all cancers at all 16 nuclear reactor locations in Germany between 1980 and 2003, including 1,592 under-fives with cancer and 4,735 controls, with 593 under-fives with leukemia and 1,766 controls. This means that the study is statistically strong and its findings statistically significant. Small numbers and weak statistical significance often limit the usefulness of many smaller epidemiological studies.

Second is its authority: it was commissioned in 2003 by the German Government's Bundesamt für Strahlenschutz (BfS, the German Federal Office for Radiation Protection, approximately equivalent to the United States EPA's Office of Air and Radiation) after requests by German citizen groups. The study was carried out by epidemiology teams from the University of Mainz which could not be accused of being opposed to nuclear power.

Third is the validity of its results, as vouchsafed for by the German Government's Bundesamt für Strahlenschutz. It officially accepted that children living near nuclear power plants develop cancer and leukemia more frequently than those living further away. It stated [9]

"The present study confirms that in Germany there is a correlation between the distance of the home from the nearest NPP [nuclear power plant] at the time of diagnosis and the risk of developing cancer (particularly leukemia) before the 5th birthday. This study is not able to state which biological risk factors could explain this relationship. Exposure to ionising radiation was neither measured nor modelled. Although previous results could be reproduced by the current study, the present status of radiobiological and epidemiological knowledge does not allow the conclusion that the ionising radiation emitted by German nuclear power stations during normal operation is the cause. This study cannot conclusively clarify whether confounders, selection or randomness play a role in the distance trend observed."

## Discussion

### (a) Other Studies on Childhood Leukemias near Nuclear Power Stations

It has been known at least since the late 1950s [10] that radiation exposures can result in increased leukemias and that environmental exposures to radiation are a risk factor for leukemia [11-13]. In addition, several ecological and case control studies [14-16] in the past have suggested or indicated an association between nuclear power plants and childhood leukemia among those living nearby.

In 1999, Laurier and Bard [17] examined the literature on childhood leukemias near nuclear power stations world-wide. They listed a total of 50 studies (29 ecological; seven case-control; and 14 national multi-site studies). The large majority revealed small increases in childhood leukemia near nuclear power stations although most of the ecological studies were not statistically significant. The policy implications of this study do not appear to have been widely discussed in the scientific media. Two studies [18,19] then indicated raised leukemia incidences in France and Germany, but official reports in the UK [20,21] and studies in France [22,23] concluded there was no evidence of leukemia increases near their respective nuclear power stations.

After the KiKK study was published in early 2008, Bithell et al [24] found a small increase in child leukemia within 0 to 5 km near 13 (of 14) UK nuclear power stations, and Laurier et al [25] found a small increase within 10 km of French nuclear power stations. In both studies, the numbers were small and therefore not considered statistically significant (i.e. there was a greater than 5% possibility that the observations could have occurred by chance).

These studies incorrectly concluded that there was "no suggestion" or "no evidence" of leukemia increases near UK and French nuclear reactors respectively. These conclusions are regrettable because low statistical significance only means that chance has not been excluded as an explanation, assuming no bias and no real effect. In more detail, p values -that is, the probabilities that observed effects may be due to chance - are affected by **both **the magnitude of the effect **and **the size of the study. This means the results of statistical tests must be interpreted with caution [26]. The difficulty is that the use of a cut-off for statistical significance (usually p = 5%) can lead to incorrectly accepting the null hypothesis (ie that there is no effect), through dismissing a result merely because it is not statistically significant (a type II error) [27]. This can occur in small studies such as Bithell et al and Laurier et al due to their small sample sizes rather than lack of effect [28,29]. In addition, weak studies which are not strong enough to pick up effects should not conclude there are no effects: that is, absence of evidence should not be construed to mean evidence of absence [30]. These are widespread misconceptions, unfortunately.

The conclusions in the Laurier et al [25] and Bithell et al [24] studies may mislead members of the public into thinking there are no increased leukemias near French or UK nuclear power stations when in fact the question remains open. The stronger evidence from the KiKK study suggests there may well be such increases - regardless of the country in which nuclear reactors are located.

Importantly, the KiKK findings are supported by a meta-analysis which combines the results of various studies in order to have large enough numbers to reach statistical significance. Baker and Hoel [31] assessed data from 17 research papers covering 136 nuclear sites in the UK, Canada, France, United States, Germany, Japan and Spain. In children up to 9 years old, leukemia death rates were from 5 to 24% higher, and leukemia incidence rates were 14 to 21% higher- see table 1. These findings were statistically significant and leant considerable support to the KiKK findings, but this study was not cited in the KiKK, Laurier and Bithell studies.

**Table 1:** Leukemia mortality risks

Age Groups	Proximity to nuclear facility	Leukemia mortality
0-9	All distances	1.05
0-9	Under 16 km	1.24
0-25	All distances	1.02
0-25	Under 16 km	1.18

Source: Baker and Hoel, 2007[31]

More recently, Dr A Körblein [32] observed the relative risk in the Bithell et al [24] data was RR=1.52 (p>0.05) and in a recent re-analysis of the KiKK data [33] using an ecological study design it was RR=1.46 (p>0.05).   Combining the 2 studies, the RR=1.49 which was statistically significant (p=0.026).

Recently, Laurier et al [34] reviewed epidemiological studies on childhood leukaemia in 198 nuclear sites in 10 countries, including 25 major multi-site studies in eight countries. They found that increased risks of childhood leukaemia near nuclear installations were a recurrent issue. They confirmed that clusters of childhood leukaemia cases existed locally, but were reluctant to generalise their findings.

### (b) Need for Powerful Epidemiological Studies

It is a truism that we should be guided by the best available scientific evidence. For a number of reasons the KiKK study provides more reliable evidence than the more recent Bithell et al [24] and Laurier et al [25] studies. First, the KiKK study found statistically significant cancer increases. The p-values in the KiKK study were 0.0034 for all cancers and 0.0044 for leukemias (both one-tailed), i.e. well below the commonly-used 0.05 figure for statistical significance. Second, the KiKK findings were supported by a meta-analysis, as mentioned above. Third, the KiKK study is a case-control study (examining 593 under five year olds with leukemia together with 1,766 controls) which means its findings should take precedence over the Bithell and Laurier studies which were less reliable ecological studies. Finally, the KIKK study used very accurate distance measures. It estimated distances between the homes of cancer cases and the chimneys of nuclear power stations to within 25 metres, unlike the imprecise areas of the Bithell and Laurier studies. The latter studies simply cannot invalidate the findings of the more sophisticated KiKK study, as their conclusions seem to imply.

### (c) KiKK Study Findings

The KiKK study showed an increased risk of cancer in children under 5 years living near all nuclear power plants in Germany. The inner 5 km zone showed an increased risk (odds ratio 1.61; lower 90% confidence limit 1.26). A categorial analysis showed a statistically significant odds ratio of 2.19 (lower 90% confidence limit: 1.51) for residential proximity within 5 km compared to residence outside this area. For all leukemias combined, the study showed a statistically significant trend for proximity to nuclear power stations with a positive regression coefficient of 1.75 [lower 90% confidence limit: 0.65]. That is, the leukemic children lived closer to nuclear power plants than randomly selected controls.

These increased risks are statistically significant and are larger than the cancer increases observed near nuclear facilities in many other countries. The data indicate that the increased risks mainly lie within 5 km of NPPs though this does not necessarily mean that there are no increased risks beyond 5 km. The most significant finding was the association between increased cancers and proximity to nuclear installations. As discussed above, many previous reports have studied increased cancer risks near nuclear facilities, but the KiKK report for the first time in Europe measured how far each cancer case was from the chimney of the nearest nuclear reactor. This allowed the study to examine the distance/risk relationship. The proximity-risk relationship was pronounced for leukemias - see table 2.

**Table 2:** KiKK odds ratios for leukemias in children < 5 years old

Distance from reactor - km	Mean distance - km	Odds ratio
>5	3	1.76
5 to <10	8	1.26
10 to <30	18	1.10
30 to <50	37	1.05
50 to <70	57	1.03
>70	74	1.02

Source: continuous regression model used by Kaatsch et al, 2008[5]

The odds in table 2 were calculated by the KiKK authors using a linear relationship between distance and relative risk (that is, RR~e^1/r^). This is uncertain as the true relationship is unknown. For example, a number of statistical tests (the sum of squared residuals and goodness of fit) indicate that a quadratic regression model (that is, RR~) fits the KiKK data better [32].

The KiKK study tested the proximity-risk relationship by examining whether confounders could have had an appreciable effect on the result. Kaatsch et al stated their results "may possibly be influenced by confounders (like social class, pesticides, factors influencing immunological factors, exposure to other ionizing radiation)". However the companion study by the same team [6] stated as regards uncontrolled confounding "no risk factors of the necessary strength for this [KIKK] effect are known for childhood cancer and specifically childhood leukemia." The KiKK team actually tried to control for these confounders in a separate analysis but there was some self-selection among the controls interviewed, meaning they might not have been representative of the study population. For this reason, the results of their confounder analysis were not presented in their published reports. However the team stated that "none of them [the confounders] changed the distance parameter by more than one standard deviation". In other words, the confounders studied by the KiKK team appear to have had little effect on the KiKK findings.

The study investigated whether the cancer increases were due to population mixing - sometimes mooted as an explanation for increased cancers near nuclear power stations. Their results suggested this was not the case but this part of the study was underpowered, statistically speaking. Therefore there could have been such an effect as absence of evidence of effect does not provide evidence of absence.

The KiKK authors also removed each nuclear power station in turn from their analyses to see if the results were dependent on the findings near one nuclear power station alone, and the answer was no. (Unfortunately, the KiKK authors have refused to release the data for each of the 16 nuclear power stations for further analyses.)

### (d) Association vs Causality

The question arises as to whether the association found by KiKK is causative: that is, are the increased cancers due to living near the reactors. In such situations, the authoritative Bradford Hill [35] tests are usually applied. The results of applying these nine tests to the KiKK study are listed in table 3.

**Table 3:** Summary of Bradford Hill test results

	Bradford Hill Guideline	Explanation	Result
1	Strength	numbers large enough not to be chance observation	yes
2	Consistency	association observed by different persons, in different places and times	yes
3	Specificity	association limited to specific people/areas/effects	yes
4	Temporality	effects occur after exposure	yes
5	Biological gradient	association has biological gradient or dose-response relationship	yes
6	Plausibility	suspected causation fits biological knowledge of the day	no
7	Coherence	suspected causation accords with natural history and biology	no
8	Experiment/animal studies	other experimental evidence available	not available
9	Analogy	similar evidence from other studies	yes

Most of the Bradford Hill tests when applied to the KiKK study support the inference of causation between increased cancers and proximity to nuclear power stations. As regards the similar tests of plausibility/coherence with existing knowledge, it is the case that the estimated radiation doses from NPP releases are too low to cause the high cancer risks near German nuclear power stations, using current dose models. Many scientists have therefore concluded that the cause of the cancer increases cannot be releases from nuclear power stations. However they fail to consider that official dose and risk estimates may be incorrect as discussed by Crouch [36] and Sumner et al [37]. (This point is further discussed below.) In other words, the current "generally known facts" as stated by Bradford Hill may be incorrect, as official dose estimates from nuclear releases could be uncertain or unreliable. If this seems implausible, Bradford Hill applies Sherlock Holmes' dictum to Watson "...when you have eliminated the impossible, whatever remains, **however improbable**, must be the truth..." (emphasis in original). The overall conclusion is that proximity of residence to German nuclear power stations is the most likely explanation for the increased cancer risks.

### (e) Possible Explanations for Increased Cancer Incidences

Various hypotheses have been put forward to explain cancer increases near nuclear installations including coincidence; a postulated virus from population-mixing (the Kinlen hypothesis [38]); the response to the lack of childhood immunity to infectious diseases (the Greaves hypothesis [39]); parental preconception irradiation (the Gardner hypothesis [40]); genetic predisposition to cancer; synergistic effects between radiation and unnamed chemicals; or combinations of these factors. Some remain little more than suggestions, others have not been supported by the KiKK study. Although some hypotheses are vigorously promoted by individuals, none commands widespread support.

Any possible explanation must be guided by the KiKK study's main finding - that the increased risks were directly linked with proximity to nuclear power plants (NPPs). Therefore it is useful to examine those aspects of the normal operation of NPPs which might result in increased exposures and risks. These include -

▪ direct radiation, i.e. gamma rays and neutrons, from reactor cores;

▪ "skyshine" radiation from reactor neutrons being reflected back to earth by N, C and O atoms;

▪ electro-magnetic radiation from power lines near NPPs;

▪ water vapour emissions from cooling towers at about half the 16 German NPPs, and

▪ radioactive releases to the environment.

The cancer increases could also be due to a combination of the above factors, as there may well be interactions between environmental exposures we are yet to understand. For example, synergistic effects may exist between radiation and chemicals may act to increase cancer risks [41,42]. Nevertheless, this is considered unlikely as synergistic effects would not exist in combination with radiation exposures from NPPs alone and not from other radiation exposures, egg from the Chernobyl plume in 1986, natural radiation and medical radiation. These latter exposures would differ for persons living at approximately the same place.

None of these aspects was explored by the KiKK study, but the estimated risks from most of them are considered to be small or non-existent. The major exception is nuclide releases from nuclear power stations which are examined next. It is noted that the KiKK study clearly had these releases in mind when it was set up. All distances to cancer cases were measured from the station chimneys, and the geographical areas monitored specifically included areas downwind from the stations.

### (f) Radioactive Releases from Nuclear Power Stations

**Figure 1:**
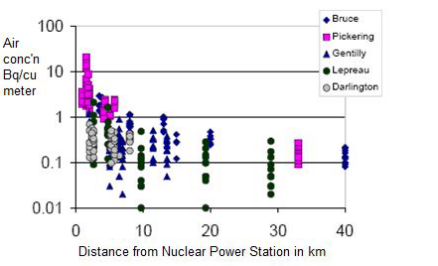
**Annual averages of tritium concentrations in air measured at distances from nuclear power stations in Canada, 1985-1999**. Abstracted from: Tritium in the Canadian Environment: Levels and Health Effects. Report RSP-0153-1 (2003). Prepared for the Canadian Nuclear Safety Commission under CNSC contract no. 87055-01-0184 by Ranasara Consultants and Richard Osborne. Data from Health Canada: Environmental Radioactivity in Canada. Radiological Monitoring Report. Ottawa, Canada: Government of Canada; 2001.

Radioactive releases from nuclear power stations occur by emissions to air and liquid discharges to rivers in Germany (or to the sea in other countries). Air emissions [43] are more important, as they cause most of the radiation dose to humans. The relationship between air releases and proximity to nuclear power stations is complicated by variable weather patterns. To say there is no relationship between releases from nuclear power stations and proximity to them would be incorrect. Figure 1 clearly shows the proximity/concentration relationship (note that the y-axis is logarithmic) near Canadian reactors. Of course, tritium air concentrations near German NPPs will be lower than those near Canadian reactors (which emit greater amounts of tritium) but the proximity/concentration relationship is likely to be similar.

When there is no wind, a simple diffusion relationship would exist in all directions from the NPP chimney. When winds occur then a relationship would exist but only in the predominant downwind direction. What should have been created by KiKK is a computer model to investigate the air releases/proximity relationship for each NPP in Germany. This would incorporate annual major nuclide releases, Pasquill weather categories, wind speeds, wind directions, and average them over a number of years, in order to estimate likely nuclide concentrations in air at the homes of cancer cases near all NPPs, and the resulting possible inhalation/ingestion doses.

The largest emissions from all pressurised water reactors (PWR) and boiling water reactors (BWR) nuclear power stations are, in order of magnitude

▪ H-3 (tritium) as radioactive water vapour

▪ C-14 as radioactive carbon dioxide, and

▪ radioactive noble gases including Kr, Ar and Xe isotopes.

**Figure 2:**
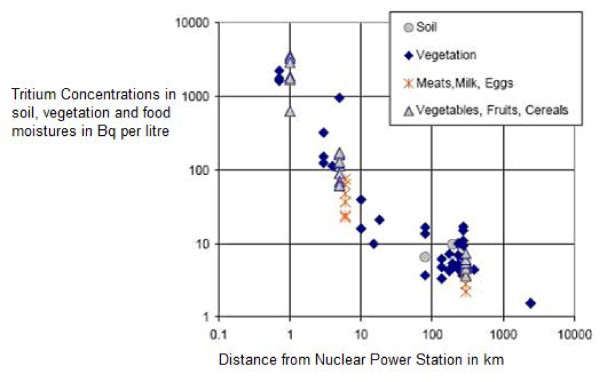
**Tritium concentrations in vegetation/food moisture near Canadian nuclear power stations**. Abstracted from: Tritium in the Canadian Environment: Levels and Health Effects. Report RSP-0153-1 (2003). Prepared for the Canadian Nuclear Safety Commission under CNSC contract no. 87055-01-0184 by Ranasara Consultants and Richard Osborne. Data from Health Canada: Environmental Radioactivity in Canada. Radiological Monitoring Report. Ottawa, Canada: Government of Canada; 2001.

These emissions result in elevated nuclide concentrations in vegetation and foodstuffs near nuclear power stations as shown in figure 2 which indicates tritium concentrations in vegetation and food moisture near Canadian nuclear power stations. This graph is log-log and indicates that (at least for distances under 20 km) the risk-proximity relationship varies approximately with 1/r^2 ^as the slope of the line is about minus 2. In other words, the tritium concentration/distance relationship resembles the risk/distance relationship observed in the KiKK study. Although tritium emissions from Canadian heavy water nuclear reactors are larger than from German PWR and BWR reactors, the same pattern of raised concentrations in vegetation and food is expected to occur near German reactors.

The most obvious explanation - releases from nuclear reactors - is often discounted because current official estimates of the radiation doses from reactor emissions are too low, typically by about three orders of magnitude, to result in the cancer risks observed by the KiKK study. But how reliable are these dose estimates and risk estimates? Unfortunately this question was not examined by the above German, UK and French studies, nor by the KiKK study itself.

### (g) Uncertainties in Dose Estimates

Estimated radiation doses to adults near nuclear power stations are invariably very low (10^-2 ^to 10^-4 ^mSv per year). How these estimates are derived is not widely understood by scientists, and not at all by members of the public. In fact, the methodology is quite complicated, as they are derived using at least four computer models in sequence

▪ models for the generation of fission/activation products in reactor cores; these generate the emission data published by utilities for most nuclides

▪ environmental transport models for radionuclides, including weather models

▪ human metabolism models which estimate nuclide uptake, retention and excretion

▪ dose models which estimate radiation doses from internally retained nuclides

Each model derives a range of results log-normally distributed from which only the median value is normally used. Each of these probability distributions would be log-normal rather than normal distributions; that is, they would be skewed to the right. This means that, although the real value could be larger or smaller than the median value, in practice some high values could result.

The problem is that each model's central result is inherently uncertain (the real result lying within the shown distribution). The uncertainties from each model have to be combined to gain an idea of the overall uncertainty in the final dose estimate [44]. Further uncertainties are introduced by unconservative radiation weighting factors and tissue weighting factors in official models [45]. The cumulative uncertainty in dose estimates could be very large as recognised by the report of the UK Government's CERRIE Committee [46].

This does not mean that official dose estimates from nuclear power plant releases are always incorrect. But it does mean they contain unquantified uncertainties which could be large and which render them unreliable where evidence exists that they may be incorrect. In other words, when we try to ascertain the reasons for the wide gulf between estimated risks and risks observed by KiKK, we should not dismiss radiation exposures as a possible cause just because official dose estimates are too low.

### (h) Uncertainties in Risk Estimates

In addition, there are uncertainties with estimated risks as well as estimated doses. This is because a risk model has to be applied to doses to estimate the likely level of cancers, but large uncertainties could exist in this model as well. For example, current official risks are derived mainly from the Japanese survivors of the atomic bombs. However many scientists worry that these risk estimates from an instantaneous external blast of high energy neutrons and gamma rays are not really applicable to the chronic, slow, internal exposures from the low-range alpha and beta radiation from most environmental releases. Uncertainties in official risk model also derive from the application of risks from a Japanese to a European population, from its application to adults only, from its application of age and gender-averaged risks, and from the practice of arbitrarily halving risks to take account of cell studies suggesting lower risks from low doses and low dose rates. However it is difficult to quantify these uncertainties and to give a figure which may indicate how much the current leukemia risk estimate may be an underestimate.

### (i) Hypothesis: In utero Exposures from Environmental Releases

The KiKK findings have prompted much debate among scientists as to the cause(s) of the increased leukemia cases near German nuclear power stations. Indeed, it is a primary task of science to attempt to explain observed phenomena which are apparently at odds with received wisdom or, in this case, with our current understanding of radiation risks. It is for this reason that the following hypothesis is suggested to explain the risks shown by the KiKK study.

It is theorised that observed high rates of infant leukemias in KiKK may be a teratogenic effect from nuclides released by nuclear reactors being incorporated in embryos and fetuses in the womb. This is suggested from the KiKK findings of increased "embryonal" cancers, that is, cancers in embryos. Spikes in releases from nuclear power stations may result in the labelling of the embryos and fetuses of pregnant women living nearby at high concentrations. These concentrations could be long-lived and could result in high doses to radiosensitive tissues and subsequent cancers. This suggestion was first made by the late Professor Edward Radford, the former Chairman of the BEIR III Committee. He mooted it 30 years ago during testimony to the Ontario Select Committee on Hydro Matters [47] which then was examining possible health effects of tritium discharges from nuclear facilities near Toronto, Canada.

**Figure 3:**
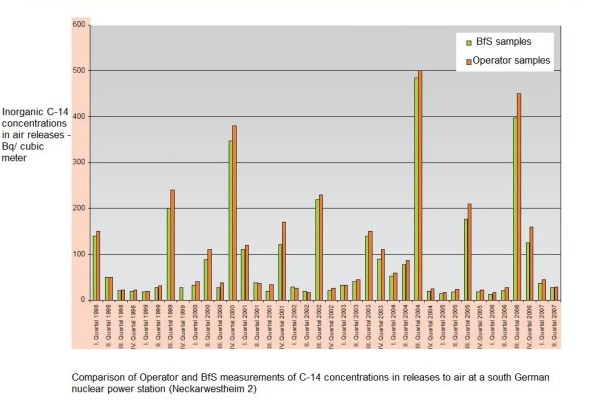
**Quarterly ^14^C air concentrations near the Neckarwestheim 2 nuclear power station in Germany**. Abstracted from Jahresbericht (Annual Yearbook) 2007, Bundesamt für Strahlenschutz, Berlin, Germany.

Spikes in the emissions of radioactive carbon and hydrogen (as carbon dioxide and water vapour) occur at nuclear power reactors when their pressure vessels are opened (approximately once a year) to replace nuclear fuel. Figure 3 indicates quarterly ^14^C releases from a German PWR nuclear power station in recent years. Tritium and noble gases will be released at the same time as ^14^C. It can be seen that gaseous releases are episodic with spikes occurring about once per year.

In order to assess this hypothesis, we discuss below a number of aspects which lend support to it, including

▪ the nature of the emissions from nuclear power stations i.e. mostly carbon (^14^C) and hydrogen (^3^H)

▪ the bio-accumulation of ^3^H and ^14^C in embryos and fetuses

▪ the increased radiosensitivity of embryos and fetuses, and

▪ the increased radiosensitivity of pre-natal hematopoietic cells

### (j) Major Radioactive Emissions: Carbon (^14^C) and Hydrogen (^3^H)

As stated above, the largest nuclide emissions from nuclear power stations are radioactive carbon (^14^C), hydrogen (^3^H) and some noble gases. ^3^H and ^14^C exist in the forms of liquid water, water vapour and carbon dioxide gas. These isotopes rapidly exchange with stable H and C; and recycle in biota. Figure 3 above indicates the relationship between tritium concentrations in food/vegetation/soil and distance from nuclear power stations. A similar relationship is expected for carbon-14.

Organically Bound Tritium (OBT) and Organically Bound Carbon (OBC) are formed by embryos and fetuses taking up tritium and ^14^C atoms during new cell production. The result is that embryos and fetuses near nuclear power stations may be labelled at the levels of environmental ^3^H and ^14^C concentrations. The resulting radiation could lead to the formation of pre-leukemic clones in the critical period of development (organogenesis) which later may lead to full leukemia.

### (k) Bio-Accumulation of ^3^H and ^14^C in Embryos And Fetuses

Stather et al [48] have estimated that, following tritium intakes by the mother during pregnancy, tritium concentrations in her fetus are 60% higher than in herself. As a result, the HPA now estimates [49] that doses in embryonic and fetal tissues are raised by factors of 1.5 to 2 compared to adult tissues following exposures to air releases of tritiated water vapour (HTO). Both studies showed similar increases for ^14^C intakes.

### (l) Increased Radiosensitivities of Embryos and Fetuses

The best data on the radiation risks of in utero exposures, that is, on the radiosensitivity of embryos and fetuses, are from the UK Oxford Survey of Childhood Cancer (OSCC) in the 1950s to 1980s [50]. Recently, Wakeford [51] comprehensively reviewed the OSCC and more than 30 similar studies worldwide. The latter studies confirmed the presence and size of the risks of in utero radiation initially found by Stewart. From OSCC and other data, Wakeford and Little [52] have estimated that the excess relative risk (ERR) of leukemia in children aged under 15 was 51 per Gy (95% CI: 28, 76) from abdominal exposures to X-rays.

If we apply this risk estimate to the KiKK situation, three corrections are needed. First, the leukemia risk rate for under 5 year-olds (as in KiKK) is greater than that for under 15 year-olds because the peak years for leukemia diagnoses are in children aged two to three years. This would result in the average relative risk being greater by a factor of perhaps ~1.5. Also, most (>90%) OSCC exposures were in the last trimester, and it has been estimated [53] that risks from exposures in the first trimester are perhaps five times greater than those from exposures in the last trimester.

These risks arose from external X-rays, whereas the KiKK risks are hypothesised to arise from internal exposures to radionuclides. There are few estimates of the risks arising from internal in utero exposures. However Fucic et al [54] have recently suggested that in utero risks from internal nuclides were four to five times greater than from in utero X-rays*. Summing these factors, we postulate that the relative risk (RR) of child leukemia in 0-5 year olds from internal nuclides in the first trimester near nuclear power stations would be about 2 per mGy. This suggests that human embryos and fetuses may be considerably more radiosensitive than currently acknowledged. It also suggests that background radiation of about 1 mGy per year (excluding radon doses) could be a major cause of naturally-occurring childhood leukemia: this has already been proposed [55].

If we were to apply the KiKK relative risk for childhood leukemia of 2.2, it would suggest in utero doses to embryos in pregnant women near German nuclear power stations of a few mGy or so. Although this is a low dose, it is still about 1,000 times higher than the official estimated doses of a few μGy (albeit to adults) from emissions from nuclear power stations.

### (m) Increased Radiosensitivity of Pre-natal Hematopoietic Cells

Finally, we need to consider the different radiosensitivities of various embryonic tissues. Since we are primarily concerned with leukemia which is a cancer of white blood cells, our attention is focussed on the hematopoietic^† ^system, i.e. bone marrow and lymphatic tissues. These contain many stem cells which create new cells: indeed, a large percentage of the stem cells in humans are found in hematopoietic tissues. Radiation-caused mutations to stem cells would clearly be damaging to the hematopoietic system and could result in increased malformation rates of white blood cells, i.e. in increased leukemia risks.

Bone marrow contains a high concentration of stem cells compared to other organs and it is likely to be among the most radiosensitive of embryonic/fetal tissues. This pronounced radiosensitivity has been remarked upon in the past. In 1990, after the Gardner team [56] had published their hypothesis of paternal preconception irradiation, the BMJ published various letters questioning the hypothesis. One by Morris [57] stated that, assuming mutations were the cause of the observed 10-fold increase in leukemia incidence observed by Gardner's team, it would require a 100 to 1,000-fold increase in the radiation-induced mutation rate if acting on the germ cell; a 10-fold increase if acting on lymphocytes during early extra-uterine life; but only a 1.8-fold increase if acting on lymphocytes throughout intrauterine life, i.e. >100 fold increase in embryo radiosensitivity. He added the latter seemed the most plausible mechanism even though the exposure pathways were unclear [58].

In 1992, Lord et al [59] made a similar suggestion when they stated that pre-natal hematopoietic cells could be up to 1,000 times more radiosensitive than post-natal ones. They added that different mechanisms of inducing this damage operated at different embryonic/fetal stages. More recently, the suggestion that pre-natal hematopoietic cells are highly radiosensitive was supported by Ohtaki et al [60] in their study of chromosome translocation frequencies in the white blood cells of Japanese A-bomb survivors irradiated in utero. They found that precursor lymphocytes of the fetal hematopoietic system may be highly radiosensitive, perhaps 100 times more so than post-natal lymphocytes. From this study, Wakeford [51] surmised that radiosensitive primitive cells (whose mutation may result in childhood cancers) remain active throughout pregnancy, including during the third trimester but not after birth, although it is not known at present why this is the case.

It is concluded that the increased radiosensitivity of hematopoietic cells before birth might prove to be a major factor in explaining the discrepancy between official dose estimates and the observed level of risks in the KiKK study.

### (n) Increases in Embryonal Cancers

Although the increased numbers of embryonal cancers in the KiKK study were not statistically significant, this does not mean that there are no such risks (see discussion above). There are good theoretical grounds for expecting solid cancers in KiKK. For example, the OSCC study [49] found increased incidences of solid cancers as well as leukemias from in utero exposures. The numerical difference between leukemia risks and solid cancer risks could be explained by the exceptional radiosensitivity of hematopoietic tissues in utero compared to other tissues. This in turn could be explained by their higher concentrations of stem cells compared with other tissues and organs.

## Conclusion

It is proposed that the observed high rates of infant leukemias in the KiKK study may be a teratogenic effect from incorporated radionuclides. Such effects, egg congenital malformations, are often recognised at birth but infant leukemia is not easily ascertained. Such babies are born pre-leukemic with full-blown leukemias only being diagnosed after birth, i.e. after their bone marrows have accumulated sufficient radioactive decays.

A possible biological mechanism to explain the KiKK observations is that emission spikes from nuclear reactors result in the radioactive labelling of embryonic and fetal tissues in pregnant women living nearby. Such concentrations, factored over two to five years both before and after birth could result in radiation exposures to the radiosensitive organs of embryos and fetuses, particularly their hematopoietic tissues. Cumulative radiation doses and risks to specific organs and tissues in embryos/fetuses from nuclide uptakes during pregnancy are not specifically considered in official publications on radiation protection.

Whatever the final explanation for the increases, the KiKK study and its implications raise many questions, including whether vulnerable people, such as pregnant women and women of child-bearing age, should be advised on possible risks of living near nuclear power stations.

It is recommended that US regulatory agencies should establish a KiKK-style epidemiological study of cancer incidences near all US nuclear power stations with precise distances being measured between cancer cases and nuclear reactors. In particular, they should establish whether a significant relationship exists between increased cancers among <5 year olds within 5 km of nuclear power stations and proximity to them. Inter alia, they should also estimate ^14^C and ^3^H uptakes to nearby residents from US nuclear power stations and from other sources. They should also estimate doses and risks from episodic nuclide emissions from nuclear power stations; estimate bone marrow doses to developing embryos and the subsequent risks of leukemia and solid cancers in very young children; assess the confidence intervals around their estimates and publish their results.

## List of Abbreviations

BWR: boiling water reactor; CERRIE (UK): Committee Examining the Radiation Risks of Internal Emitters; CI: confidence interval; COMARE (UK): Committee on the Medical Aspects of Radiation in the Environment; ERR: excess relative risk; KiKK: Kinderkrebs in der Umgebung von Kernkraftwerken = Childhood Cancer in the Vicinity of Nuclear Power Plants; mGy: milligrays; mSv: millisieverts; NPP: nuclear power plant; OBC: organically bound carbon; OBT: organically bound tritium; OSCC (UK): Oxford Survey of Childhood Cancer; PWR: pressurised water reactor; RR: relative risk; μGy: micrograys.

## Competing interests

The author declares that he has no competing interests.

## Note

*the internal nuclides studied by Fucic et al were mainly ^99m^Tc and ^131^I.

^†^hematopoiesis - sometimes termed hemopoiesis - is the formation of blood cellular components.
